# Complement components are upregulated and correlate with disease progression in the TDP-43^Q331K^ mouse model of amyotrophic lateral sclerosis

**DOI:** 10.1186/s12974-018-1217-2

**Published:** 2018-06-01

**Authors:** John D. Lee, Samantha C. Levin, Emily F. Willis, Rui Li, Trent M. Woodruff, Peter G. Noakes

**Affiliations:** 1 School of Biomedical Sciences, the University of Queensland, St Lucia, Brisbane, QLD 4072 Australia; 2 University of Queensland Centre for Clinical Research, the University of Queensland, Herston, Brisbane, QLD 4029 Australia; 3 Queensland Brain Institute, the University of Queensland, St Lucia, Brisbane, QLD 4072 Australia

## Abstract

**Background:**

Components of the innate immune complement system have been implicated in the pathogenesis of amyotrophic lateral sclerosis (ALS) specifically using hSOD1 transgenic animals; however, a comprehensive examination of complement expression in other transgenic ALS models has not been performed. This study therefore aimed to determine the expression of several key complement components and regulators in the lumbar spinal cord and tibialis anterior muscle of TDP-43^Q331K^ mice during different disease ages.

**Methods:**

Non-transgenic, TDP-43^WT^ and TDP-43^Q331K^ mice were examined at three different ages of disease progression. Expression of complement components and their regulators were examined using real-time quantitative PCR and enzyme-linked immunosorbent assay. Localisation of terminal complement component receptor C5aR1 within the lumbar spinal cord was also investigated using immunohistochemistry.

**Results:**

Altered levels of several major complement factors, including C5a, in the spinal cord and tibialis anterior muscle of TDP-43^Q331K^ mice were observed as disease progressed, suggesting overall increased complement activation in TDP-43^Q331K^ mice. C5aR1 increased during disease progression, with immuno-localisation demonstrating expression on motor neurons and expression on microglia surrounding the regions of motor neuron death. There was a strong negative linear relationship between spinal cord C1qB, C3 and C5aR1 mRNA levels with hind-limb grip strength.

**Conclusions:**

These results indicate that similar to SOD1 transgenic animals, local complement activation and increased expression of C5aR1 may contribute to motor neuron death and neuromuscular junction denervation in the TDP-43^Q331K^ mouse ALS model. This further validates C5aR1 as a potential therapeutic target for ALS.

## Background

Amyotrophic lateral sclerosis (ALS), also known as motor neuron disease, is a devastatingly fatal neurodegenerative disorder for which there are few effective treatments. ALS is characterised by loss of cortical spinal neurons of the motor cortex and alpha motor neurons within the brainstem and spinal cord, which results in skeletal muscle atrophy and progressive paralysis, eventually leading to death within 2 to 5 years of diagnosis. In the vast majority of ALS patients (~ 90%), the disease develops sporadically; however, in a minority of cases (~ 10%), the disease has a familial component, and it is due to specific genetic mutations. Some of the genes that have been implicated in ALS include *C9orf72*, *VCP*, *FUS*, *SOD1* and *TARDBP* (TDP-43) [1–5]. Despite these differing aetiologies, sporadic and familial ALS patients are clinically and pathologically indistinguishable, suggesting that regardless of whether an ALS patient carries a known ALS mutation, or is sporadic, the underlying mechanism of motor neuron dysfunction is similar [6]. Numerous mechanisms have been proposed to contribute to ALS pathophysiology, including neuroinflammation. A key mediator of neuroinflammation is the chronic activation of the complement system, proposed to drive ALS disease progression [7].

Multiple clinical and experimental studies have shown compelling evidence that complement activation is involved in the pathogenesis of ALS, whereby components of all the complement pathways are upregulated in the serum, cerebrospinal fluid, skeletal muscles and neurological tissue (spinal cord and motor cortex) of ALS patients, as well as in transgenic SOD1 animal models of ALS [8]. Chronic complement activation is proposed to drive ALS disease progression through the actions of the pro-inflammatory complement peptide, C5a, signalling through its main receptor C5aR1 [9, 10]. This pathogenic role of C5a-C5aR1 is proposed to drive disease progression through inducing glial chemotaxis, activation of local immune cells and infiltration of macrophages into skeletal muscles, thereby inducing an overall increase in inflammation/neuroinflammation and thus neurodegeneration [9–12]. However, the pathogenic role of C5a-C5aR1 signalling in ALS has primarily been shown in transgenic SOD1 rodent models of ALS [9, 12]. Hence, it is unknown whether C5a-C5aR1 pathogenic signalling is specific to ALS cases not characterised by SOD1 pathology. Thus, as majority of ALS patients show TDP-43 pathology (~ 95%), the current study aimed to investigate complement in a recently developed TDP-43^Q331K^ mouse model of ALS [13].

We examined the expression of major complement factors and of C5a and its receptor C5aR1, within the lumbar spinal cord and tibialis anterior (TA) leg muscle at three different ages during disease progression in TDP-43^Q331K^ mice, in order to provide a comprehensive overview of the potential involvement of complement in an alternative mouse model of ALS. Our findings demonstrate that a global dysregulation of complement system is involved in this TDP-43 familial mouse model of ALS, suggesting that complement/C5aR1 could be a potential therapeutic target in most forms of ALS.

## Methods

### Animals

Transgenic TDP-43^WT^ (Line 96) and TDP-43^Q331K^ (Line 103) mice were obtained from the Jackson Laboratory (Bar Harbor, ME, USA) and were bred on a C57BL/6J background to produce TDP-43^WT^, TDP-43^Q331K^ and respective non-transgenic (NTg) control mice. TDP-43^WT^ transgenic mice express a myc-tagged human non-mutated version of the TDP-43 cDNA sequence and TDP-43^Q331K^ mice express a myc-tagged, human TDP-43 cDNA modified to have an ALS-linked glutamine to lysine residue mutation at position 331, under the direction of the mouse prion protein promoter. The prion protein promoter ensures that the transgene expression is directed primarily to the central nervous system—the brain and spinal cord—and is very low in other peripheral tissues [13]. Female NTg, TDP-43^WT^ and TDP-43^Q331K^ mice at 3, 10 months and 16 months based on their motor deficits were used in this study [14]. TDP-43^WT^ mice have moderate overexpression levels in total TDP-43, with a 1.5-fold increase in total TDP-43 expression and similar levels of human wild-type TDP-43 levels compared with endogenous TDP-43 in NTg mice. TDP-43^Q331K^ mice also have moderate overexpression levels in total TDP-43, with a 2.5-fold increase in total TDP-43 expression and 1.5-fold greater expression of human Q331K TDP-43 levels compared with endogenous TDP-43 in NTg mice [13].

### Hind-limb grip strength test

The hind-limb grip strength of NTg, TDP-43^WT^ and TDP-43^Q331K^ female mice (*n* = 15 per genotype) was measured by using a grip strength meter (IMADA, Toyohashi, Japan) at 3, 10 and 16 months of age at the same time (14:00 h) of the day as previously described by us [9, 15, 16]. In brief, mice were held by their tails and lowered until they grasped the T-bar connected to the digital force transducer with their hind-limbs. The tail was lowered until the body was horizontal, and the mouse was pulled away from the T-bar with a smooth steady pull until both hind-limbs released the T-bar. The strength of the grip was measured in newtons. Each mouse was given 10 attempts, and the maximum strength was recorded [9].

### Tissue preparation for microglia/astrocyte quantification and immunohistochemistry

Female NTg, TDP-43^WT^ and TDP-43^Q331K^ mice (*n* = 4 per genotype) were euthanized by intraperitoneal injection of zolazapam (50 mg/kg; Zoletil, Lyppard) and xylazine (10 mg/kg; Xylazil, Lyppard). Mice were then fixed by transcardiac perfusion with 2% sodium nitrite in 0.1 M phosphate buffer (pH 7.4; Sigma-Aldrich, St Louis, MO, USA) followed by 4% paraformaldehyde in 0.1 M phosphate buffer (4% PFA-PB; pH 7.4; Sigma-Aldrich, St Louis, MO, USA) at the previously mentioned ages. Lumbar spinal cords were collected and placed into 4% PFA-PB for 2 h at 4 °C. Following this incubation, the spinal cords were washed 3 × 5 min in phosphate-buffered saline (PBS; pH 7.4), followed by submersion in sucrose solution at 15% then 30% in PBS (pH 7.4). Lumbar spinal cords were then embedded in optimal cutting temperature compound (Sakura, Finetek, Torrance, CA, USA) then snap frozen in liquid nitrogen. Lumbar spinal cords were sectioned into 16-μm-thick transverse and coronal sections and dry mounted onto Superfrost Plus slides (Menzel-Glaser, Braunschweig, Germany) for estimation of astrocytes, microglia and immunohistochemistry as detailed below.

### Estimation of astrocytes and microglia

For estimation of astrocytes and microglia within the lumbar spinal cord, sections were rehydrated in PBS (pH 7.4) then blocked in PBS containing 3% bovine serum albumin (BSA) for 1 h at room temperature. Sections were incubated overnight at 4 °C with the astrocyte (mouse anti-GFAP; 1:1000, BD Biosciences, San Diego, CA, USA) and microglia (rat anti-CD11b; 1:500, Abcam, Cambridge, MA, USA) markers. Sections were washed with PBS for 3 × 10 min prior to incubation overnight at 4 °C with the Alexa secondary cocktail: Alexa Fluor 555 dye-conjugated goat anti-rat (1:1000, Invitrogen, Eugene, OR, USA) and Alexa Fluor 488 dye-conjugated goat anti-mouse (1:600, Invitrogen, Eugene, OR, USA) antibody. All primary and secondary antibodies were diluted in PBS (pH 7.4) containing 1% BSA. Sections were then washed for 3 × 5 min in PBS, then mounted with Prolong Gold Anti-Fade medium containing 4, 6-diamidino-2-phenylindole (DAPI; Invitrogen, Eugene, OR, USA). Quantification of GFAP and CD11b was performed on ~ 11 to 14 lumbar spinal cord sections spaced 320 μm apart and expressed as the percentage immunoreactive area per section [12]. Quantification was within the second lumbar dorsal root ganglia (L2) to the fifth lumbar dorsal root ganglia (L5), selected with the aid of the mouse spinal cord atlas [17]. Staining procedures and image exposures were all standardised between genotypes and between sections. The mouse genotype was not made available to the researchers until the completion of the study.

### Quantification of activated microglia numbers

The cell body of microglia was labelled with the nuclear marker, DAPI. As microglia are known to display morphological changes when they become activated, such as an increase in cell body size, thickening of proximal processes and a decrease in the ramification of distal branches [18], activated microglia were defined by the presence of one DAPI stain, an amoeboid cell body and proximal processes length ≤ 1–2 μm [19]. The total number of activated microglia was determined by the average of 11–14 sections, with the overall average multiplied by the number of sections within L2–L5. The mouse genotype was not made available to the researchers until quantification was completed.

### Immunohistochemistry

Fluorescence double-labelling immunohistochemistry was performed to localise C1q and C5aR1 expression with specific cell-type markers for astrocytes, microglia and motor neurons as previously described [15]. Briefly, sections were rehydrated in PBS (pH 7.4) for 10 min, then blocked in PBS containing 3% BSA or 3% donkey serum (DS) for 1 h at room temperature. Sections were incubated overnight at 4 °C with combination of primary antibodies outlined in Table 1. All primary antibodies were diluted in PBS (pH 7.4) containing 1% BSA or 1% DS. Sections were washed 3 × 10 min with PBS prior to incubation with an appropriate Alexa-conjugated secondary cocktail: Alexa 555 goat anti-rat, Alexa 594 donkey anti-rat, Alexa 488 goat anti-mouse, Alexa 488 goat anti-rabbit and Alexa 488 donkey anti-goat (Invitrogen, Eugene, OR, USA). All secondary antibodies were diluted in PBS (pH 7.4) containing 1% BSA or 1% DS (1:1,1000 for Alexa 555, 1:500 for Alexa 594 and 1:600 for Alexa 488). Following 3 × 5 min washes in PBS, all sections were mounted with Prolong Gold Anti-Fade medium containing DAPI (Invitrogen, Eugene, OR, USA). Sections with no primary antibodies were used as negative controls for all immunohistochemistry experiments to give a measure of non-specific background staining.

**Table 1:** Summary of antibodies used for immunohistochemistry

Antibody	Manufacturer	Dilution	In combination with one of the following antibodies
Rat anti-mouse C5aR1	Bio-Rad	1:250	GFAP, Iba-1 or ChAT
Rat anti-mouse C1q	Hycult Biotechnology	1:1000	GFAP, Iba-1 or ChAT
Goat anti-mouse ChAT	Merck	1:100	C1q or C5aR1
Rabbit anti-mouse Iba-1	Wako	1:400	C1q or C5aR1
Mouse anti-rat GFAP	BD Biosciences	1:1000	C1q or C5aR1

Quantification of immunofluorescence for C1q was performed on ~ 25 to 35 lumbar spinal cord sections (per animal; *n* = 4) spaced 160 μm apart and expressed as the percentage immunoreactive area per section. Staining procedures and image exposures were all standardised between genotype and between sections [15].

### Real-time quantitative PCR

Total RNA was isolated from the spinal cord and TA muscle of NTg, TDP-43^WT^ and TDP-43^Q331K^ mice using RNeasy Lipid Tissue extraction kit according to manufacturer’s instructions (QIAGEN, CA, USA). Total RNA was purified from genomic DNA contamination using Turbo DNAse treatment (Ambion, NY, USA), then converted to cDNA using AffinityScript cDNA synthesis kit according to manufacturer’s instructions (Agilent Technologies, CA, USA). Commercially available gene-specific Taqman probes for complement component 1, q subcomponent, beta polypeptide (C1qB; Mm01179619_m1), complement component 4 (C4; Mm00437893_g1), complement factor B (Cfb; Mm00433909_m1), complement component 3 (C3; Mm01232779_m1), CD55 antigen (Cd55; Mm00438377_m1), CD59a antigen (Cd59a; Mm00483149_m1) and complement component 5a receptor 1 (C5ar1; Mm00500292_s1) were used to amplify target gene of interest (Applied Biosystems, MA, USA). Relative target gene expression to geometric mean of reference genes glyceraldehyde-3-phosphate dehydrogenase (Gapdh; Mm99999915_g1), beta actin (Actb; Mm02619580_g1) and hypoxanthine guanine phosphoribosyl transferase (Hprt; Mm03024075_m1) was determined using this formula: 2^-∆CT^ where ∆CT = (Ct _(Target gene)_ − Ct _(Gapdh, Actb and Hprt)_), as per our previous studies [15, 16]. Final measures are presented as relative levels of gene expression in TDP-43^WT^ and TDP-43^Q331K^ mice compared with expression in NTg controls. Probe sets were tested over a serial cDNA concentration for amplification efficiency. No reverse transcription and water as no template control was used as negative controls. All samples were run in triplicate and were tested in three separate experiments.

### Enzyme-linked immunosorbent assay

Ninety-six-well plates (Greiner Bio-One, Frickenhausen, Germany) were pre-coated with rat anti-mouse C5a capture antibody (4 μg/mL; R&D Systems, Minneapolis, MN, USA) diluted in PBS (pH 7.4) overnight at room temperature in a sealed humidified container. Following the plate being blocked for 1 h at room temperature with reagent diluent (1% BSA in PBS), C5a standard, spinal cord and TA muscle homogenates were incubated for 2 h at room temperature. The plates were subsequently incubated with biotinylated goat anti-mouse C5a detection antibody (0.2 μg/mL, R&D Systems, Minneapolis, MN, USA) for 1 h at room temperature, and then incubated with Streptavidin-HRP conjugate for 20 min at room temperature. Tetramethylbenzidine (Sigma-Aldrich, Saint Louis, MO, USA) substrate was used as the chromogen, and the plate was read at 450 nm. Levels of C5a in spinal cord and TA muscle samples were adjusted to micrograms per protein and expressed as nanograms of C5a per microgram of protein.

### Statistical analysis

All statistical analyses were performed using GraphPad Prism 7.0 (GraphPad Software Inc., San Diego, CA, USA). For the results from GFAP and CD11b quantification, quantitative real-time PCR and enzyme-linked immunosorbent assay, statistical differences between NTg, TDP-43^WT^ and TDP-43^Q331K^ mice were determined using one-way ANOVA with Tukey’s post hoc test for each age group. To assess the linear association between complement mRNA transcript levels and hind-limb grip strength of NTg, TDP-43^WT^ and TDP-43^Q331K^ mice, Pearson’s correlation was used. All data was presented as mean ± standard error of mean and differences considered significant when *P* < 0.05.

## Results

### Motor deficits in TDP-43^Q331K^ mice correlate with increases in astrocytes and microglia during disease progression

**Fig. 1:**
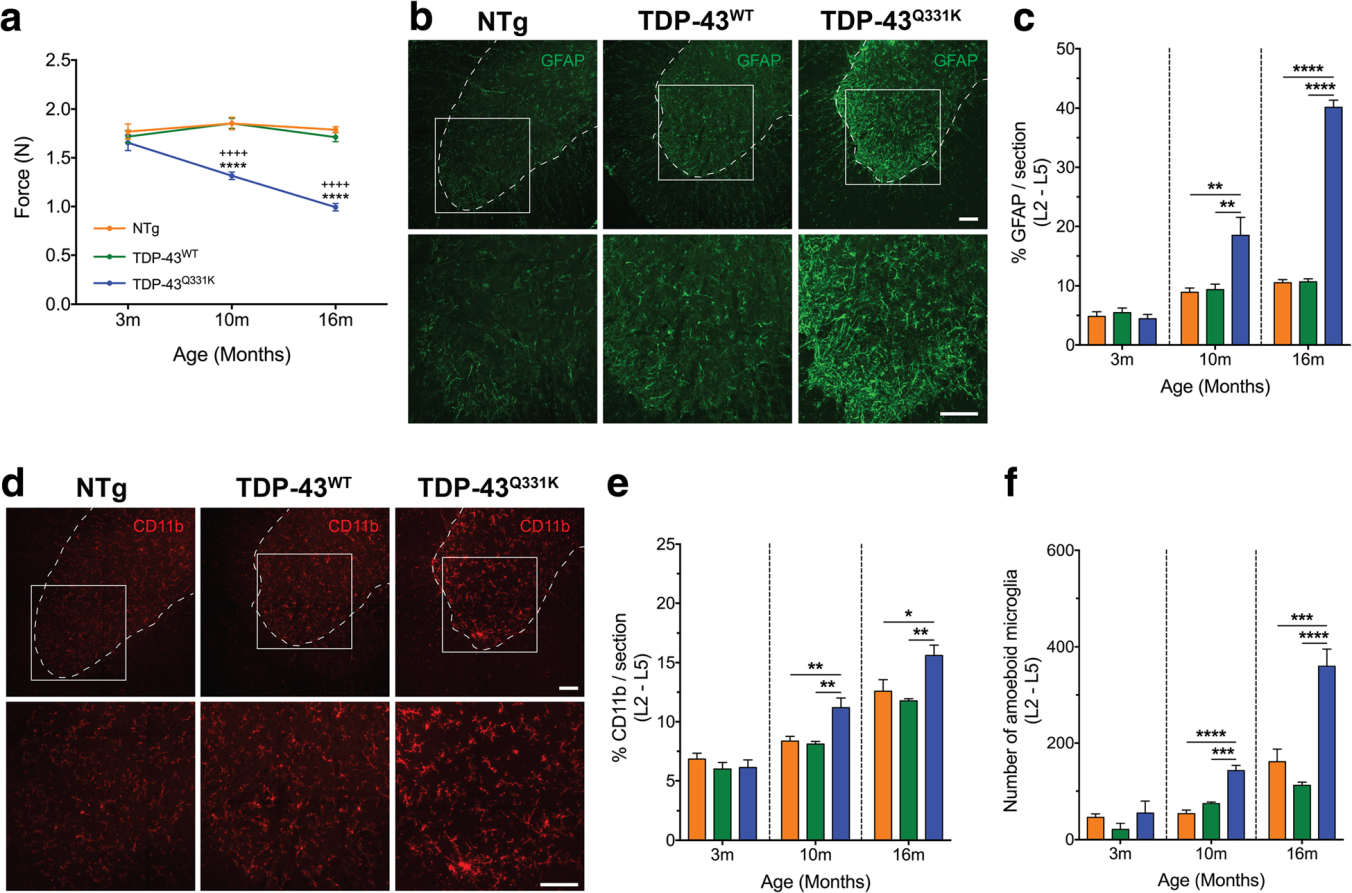
Decline in hind-limb grip strength during ALS progression correlates with increase in astrocytes and microglia in the lumbar spinal cord of TDP-43^Q331K^ mice. **a** Hind-limb grip strength (N) from NTg, TDP-43^WT^ and TDP-43^Q331K^ mice at 3, 10 and 16 months of age. At 3 months, no significant difference in force is present between genotypes. By 10 months, TDP-43^Q331K^ mice display a significant decrease in hind-limb grip strength compared with both NTg and TDP-43^WT^ mice (~ 30% reduction; NTg vs TDP-43^Q331K^ ****P < 0.0001 and TDP-43^WT^ vs TDP-43^Q331K^ ++++*P* < 0.0001; *n* = 15). A progressive decline in hind-limb grip strength is present at 16 months, with TDP43^Q331K^ mice showing significantly lower hind-limb grip strength compared with NTg and TDP-43^WT^ mice (~ 45% reduction; NTg vs TDP-43^Q331K^ ****P < 0.0001 and TDP-43^WT^ vs TDP-43^Q331K^ ++++*P* < 0.0001; *n* = 15). **b** Representative images of the GFAP-positive astrocytes in the lumbar spinal cord of NTg, TDP-43^WT^ and TDP-43^Q331K^ animals at 16 months. Dashed line shows the outline of the ventral horn with higher magnification of the white square. Scale bars = 100 μm. **c** Increased astrocyte expression in TDP-43^Q331K^ mice (blue bars) compared with NTg (orange bars) and TDP-43^WT^ mice (green bars) at ages 10 and 16 months (**P < 0.01, ****P < 0.0001; *n* = 4). **d** Representative images of CD11b-positive microglia in the lumbar spinal cord of NTg, TDP-43^WT^ and TDP-43^Q331K^ animals at 16 months. Dashed line shows the outline of the ventral horn with higher magnification of the white square. Scale bar = 100 μm. **e** Increased microglia expression in TDP-43^Q331K^ mice (blue bars) compared with NTg (orange bars) and TDP-43^WT^ mice (green bars) at ages 10 and 16 months (*P < 0.05, **P < 0.01; *n* = 4). **f** Increased number of activated microglia (amoeboid) in TDP-43^Q331K^ mice (blue bars) compared with NTg (orange bars) and TDP-43^WT^ (green bars) mice at 10 and 16 months (***P < 0.001, ****P < 0.0001; *n* = 4). Data are presented as mean ± SEM; one-way ANOVA with Tukey’s post hoc test for each age

To monitor the decline in motor performance during disease onset and progression in TDP-43^Q331K^ mice, we performed hind-limb grip strength tests in animals. At 3 months, there was no difference in hind-limb grip strength between NTg, TDP-43^WT^ and TDP-43^Q331K^ mice (Fig. 1a). However, by 10 months, we observed a significant reduction in hind-limb grip strength in TDP-43^Q331K^ mice when compared with NTg and TDP-43^WT^ mice (~ 30% reduction, *n* = 15, ****P < 0.0001, ++++*P* < 0.0001; Fig. 1a). Furthermore, at 16 months, there was a progressive decline in hind-limb grip strength in TDP-43^Q331K^ mice (~ 45% reduction, *n* = 15, ****P < 0.0001, ++++*P* < 0.0001; Fig. 1a) when compared with NTg and TDP-43^WT^ mice. Importantly, we found that the decline in hind-limb grip strength in TDP-43^Q331K^ mice closely correlated with the increase in immunoreactive area of astrocytes using immunofluorescence staining in the lumbar spinal cords at 10 months (~ 200% increase, *n* = 4, **P < 0.01; Fig. 1b, c) and 16 months (~ 380% increase, *n* = 4, ****P < 0.0001; Fig. 1b, c). Similarly, we also found that the decline in hind-limb grip strength in TDP-43^Q331K^ mice closely correlated with the increase in immunoreactive area of microglia and number of activated microglia in the lumbar spinal cord at 10 months (140~230% increase, *n* = 4, **P < 0.01, ***P < 0.001, ****P < 0.0001; Fig. 1d–f) and 16 months (130~280% increase, *n* = 4, *P < 0.05, **P < 0.01, ***P < 0.001, ****P < 0.0001; Fig. 1d–f). Taken together, these data reveal an age-related decline in hind-limb grip strength of TDP-43^Q331K^ mice associated with increased glial activation in the lumbar spinal cord.

### Components of the classical/lectin pathways of complement are upregulated along with decreased expression levels of complement regulator CD55 in TDP-43^Q331K^ mice

The complement system is part of the innate immune system that can contribute to neuroinflammation in many neurodegenerative diseases, including ALS [8]. Previous studies, including our own, have identified major complement components are upregulated in the lumbar spinal cord of hSOD1^G93A^ mice [15]. However, there is no comprehensive overview of complement system in different animal models of ALS other than hSOD1 transgenic mice [8]. Therefore, we measured the mRNA levels of key components of the classical/lectin pathway (C1qB and C4), alternative pathway (fB), the central component to all pathways (C3) and the complement regulators (CD55 and CD59a) in the lumbar spinal cord of NTg, TDP-43^WT^ and TDP-43^Q331K^ mice using quantitative real-time PCR during disease progression of ALS (3, 10 and 16 months).

**Fig. 2:**
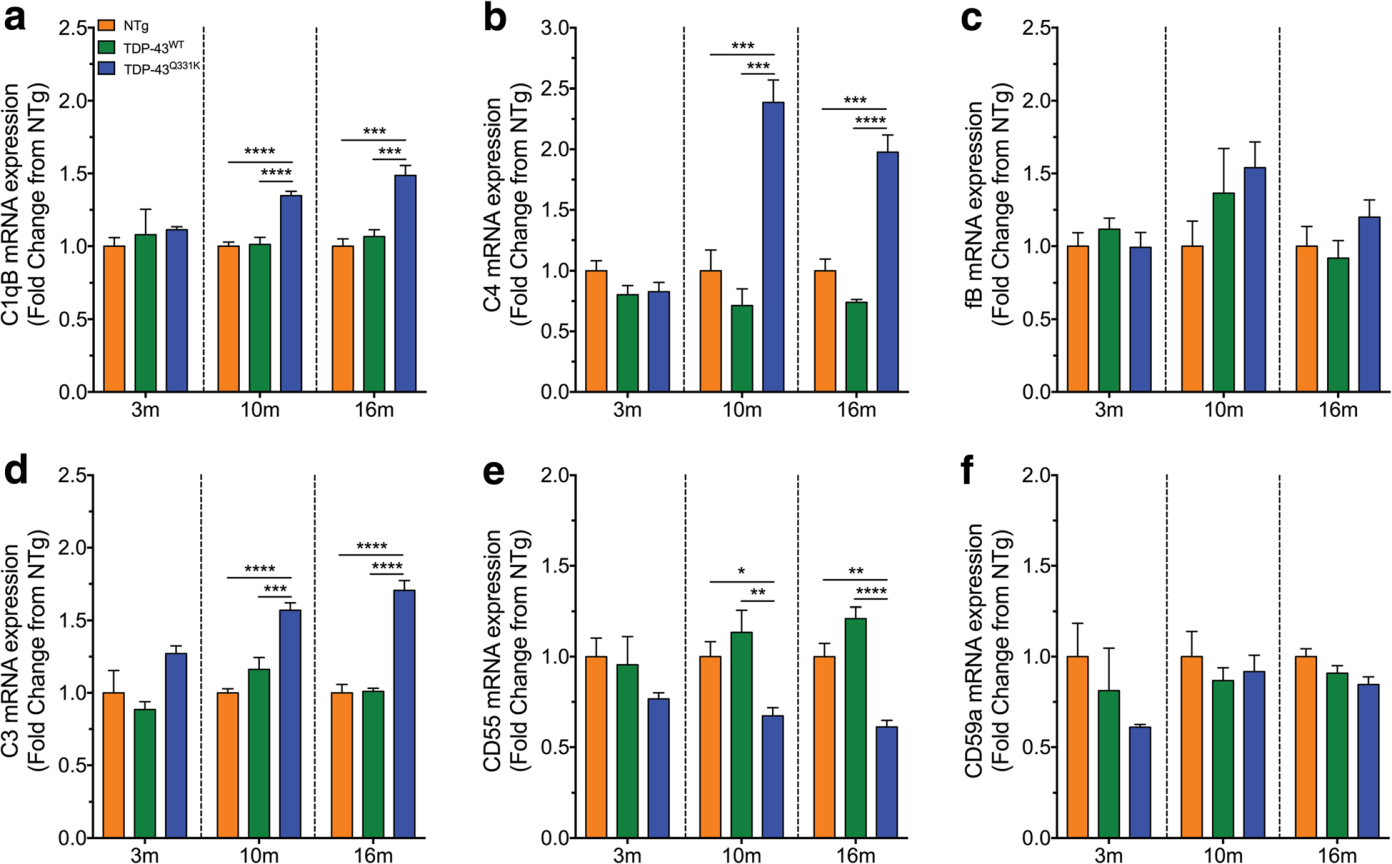
Dysregulation of complement components in the lumbar spinal cord of TDP-43^Q331K^ mice at three different ages of disease progression. **a**–**f** The mRNA expression profiles of the following complement components: C1qB (**a**, classical pathway), C4 (**b**, classical/lectin pathway), fB (**c**, alternative pathway), C3 (**d**, central component), CD55 (**e**, regulator) and CD59a (**f**, regulator) in the lumbar spinal cord of TDP-43^Q331K^ mice (blue bars) relative to non-transgenic (NTg, orange bars) and TDP-43^WT^ (green bars) mice during 3, 10 and 16 months of age. Data are expressed as means ± SEM (*n* = 5 mice/group, *P < 0.05, **P < 0.01, ***P < 0.001, ****P < 0.0001, one-way ANOVA with Tukey’s post hoc test)

Quantitative real-time PCR analyses showed significant increases of the C1qB transcript in TDP-43^Q331K^ mice by 1.3-fold at 10 months of age when compared to NTg and TDP-43^WT^ mice; at 16 months of age, the increase was 1.5-fold and 1.4-fold when compared to NTg and TDP-43^WT^ mice, respectively (blue bar compared to orange and green bars respectively in Fig. 2a; *n* = 5, ***P < 0.001, ****P < 0.0001). C4 transcript was also increased by 2.4-fold and 3.3-fold at 10 months of age and 2.0-fold and 2.7-fold at 16 months of age when compared to NTg and TDP-43^WT^ mice, respectively (blue bar compared to orange and green bars respectively in Fig. 2b; *n* = 5, ***P < 0.001, ****P < 0.0001). By contrast, fB did not show any significant changes at 10 and 16 months of age in TDP-43^Q331K^ mice when compared to NTg and TDP-43^WT^ mice (blue bar compared to orange and green bars respectively in Fig. 2c; *n* = 5, *P* > 0.05). The central component of complement, C3, was also increased in the lumbar spinal cord of TDP-43^Q331K^ mice, with a 1.6-fold and 1.4-fold increase at 10 months of age when compared to NTg and TDP-43^WT^ mice, respectively; at 16 months of age, the increase was 1.7-fold when compared to both genotypes (blue bar compared to orange and green bars respectively in Fig. 2d; *n* = 5, ***P < 0.001, ****P < 0.0001).

The negative regulators of the complement system, CD55 and CD59a, were also investigated due to their importance in maintaining homeostasis and keeping the complement system in its proper physiological state in response to altered physiological conditions (i.e. infection and/or neurodegeneration). CD55 mRNA expression in TDP-43^Q331K^ mice was decreased at 10 months of age by 0.3-fold and 0.4-fold, and by 0.4-fold and 0.5-fold at 16 months of age, when compared with NTg and TDP-43^WT^ mice (blue bar compared to orange and green bars respectively in Fig. 2e; *n* = 5, *P < 0.05, **P < 0.01, ****P < 0.0001). By contrast, CD59a mRNA expression did not significantly alter at 10 and 16 months of age in TDP-43^Q331K^ mice when compared to NTg and TDP-43^WT^ mice (blue bar compared to orange and green bars respectively in Fig. 2f; *n* = 5, *P* > 0.05). These results suggest widespread complement perturbation occurs in the lumbar spinal cord of TDP-43^Q331K^ mice, which may contribute to glial activation and neuroinflammation, and ultimately disease progression in this model.

**Fig. 3:**
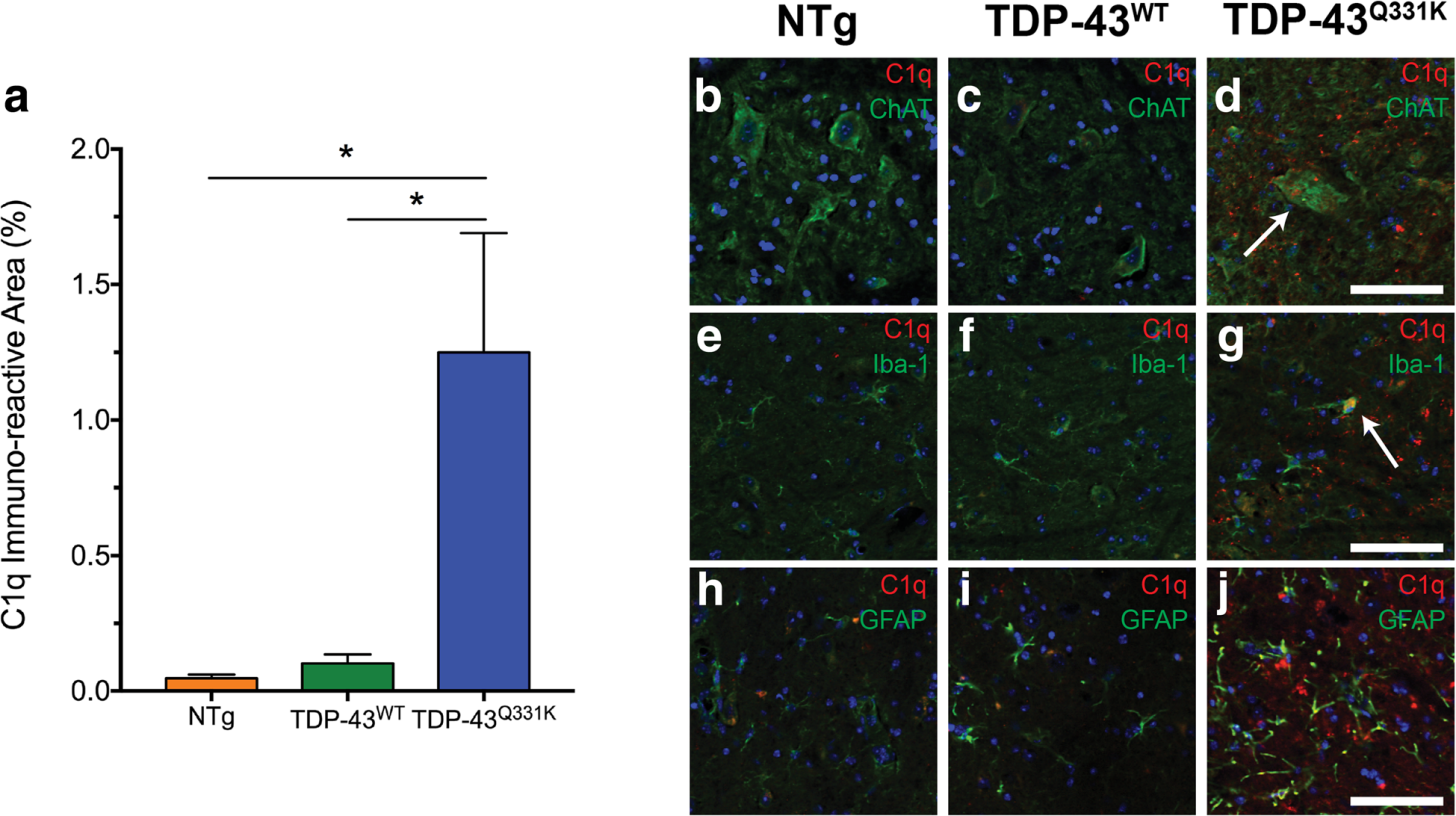
Expression and localisation of C1q in the spinal cord of non-transgenic, TDP-43^WT^ and TDP-43^Q331K^ mice at 16 months of age. **a** The immunoreactive area of C1q in the spinal cord of non-transgenic (NTg, orange bars), TDP-43^WT^ (green bars) and TDP-43^Q331K^ (blue bars) at 16 months. Data are expressed as means ± SEM (*n* = 4 mice/group, *P < 0.05, one-way ANOVA with Tukey’s post hoc test). **b**–**j** Double immunolabelling of C1q (red) with cellular markers (green) for motor neurons (ChAT; **b**–**d**), microglia (Iba-1; **e**–**g**) and astrocyte (GFAP; **h**–**j**) in the ventral lumbar spinal cord of NTg, TDP-43^WT^ and TDP-43^Q331K^ mice at 16 months of age. C1q was co-localised with ChAT-positive motor neurons (**d**, white arrow) and microglia (**g**, white arrow) in TDP-43^Q331K^ mice. Note that white arrows indicate red and green fluorescent signal merge to orange. In TDP-43^Q331K^ mice, immunolabelling of C1q was also evident on other cell types, indicated by lack of co-localisation with anti-ChAT, anti-Iba-1 and anti-GFAP (**d**, **g**, **j**). Scale bars for all panels = 20 μm

Upregulation of C1q at 16 months of age was also confirmed using immunofluorescence, where there was marked increase in TDP-43^Q331K^ mice compared with NTg and TDP-43^WT^ mice (blue bar compared to orange and green bars in Fig. 3a; *n* = 4, *P < 0.05). We also observed that the marked increase of C1q in TDP-43^Q331K^ mice was localised to motor neurons and microglia (white arrows in Fig. 3d, g), compared with NTg and TDP-43^WT^ mice where little to no C1q was observed (Fig. 3b, c, e, f). We did not observe C1q on astrocytes in either NTg, TDP-43^WT^ or TDP-43^Q331K^ mice (Fig. 3h–j).

### The terminal complement pathway C5a receptor, C5aR1, is upregulated and expressed on motor neurons and microglia in the lumbar spinal cord of TDP-43^Q331K^ mice

**Fig. 4:**
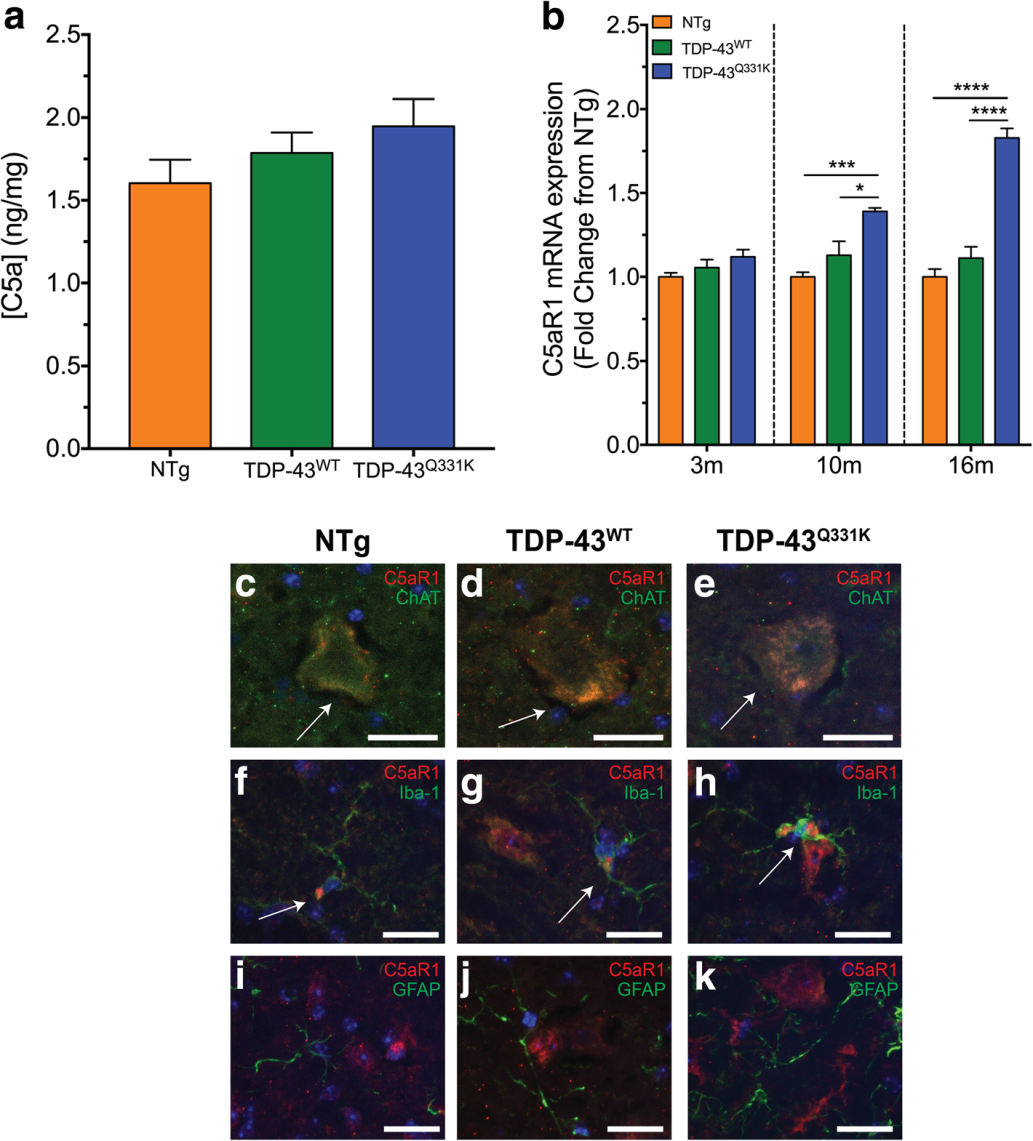
Expression of C5a and C5aR1 in the spinal cord of TDP-43^Q331K^ mice at three different ages of disease progression. **a** The protein expression of C5a in the spinal cord of non-transgenic (NTg, orange bars), TDP-43^WT^ (green bars) and TDP-43^Q331K^ (blue bars) at 16 months. **b** mRNA expression of C5aR1 in the spinal cord of TDP-43^Q331K^ mice relative to age-matched NTg and TDP-43^WT^ mice at 3, 10 and 16 months of age. Data are expressed as means ± SEM (*n* = 5 mice/group, *P < 0.05, ***P < 0.001, ****P < 0.0001, one-way ANOVA with Tukey’s post hoc test for each age). **c**–**k** Double immunolabelling of C5aR1 (red) with cellular markers (green) for motor neurons (ChAT; **c**–**e**), microglia (Iba-1; **f**–**h**) and astrocyte (GFAP; **i**–**k**) in the ventral lumbar spinal cord of NTg, TDP-43^WT^ and TDP-43^Q331K^ mice at 16 months of age. C5aR1 was co-localised with ChAT-positive motor neurons (**c**–**e**, white arrows) and microglia (**f**–**h**, white arrows) in NTg, TDP-43^WT^ and TDP-43^Q331K^ mice. Note that white arrows indicate red and green fluorescent signal merge to orange. Scale bars for all panels = 20 μm

Previous studies have shown increases in C5aR1 expression in the central nervous system of multiple rodent models of ALS, with many studies suggesting a pathogenic role for C5aR1 in the disease progression of ALS in hSOD1^G93A^ mice [9, 10, 12, 20]. C5a, the ligand for C5aR1, is an activation fragment of the terminal complement cascade that is rapidly generated following complement cascade initiation [21]. We therefore examined the protein levels of C5a in the spinal cord of TDP-43^Q331K^ mice using enzyme-linked immunosorbent assay as a biomarker for terminal complement activation. Interestingly, the results showed no change in C5a protein levels in TDP-43^Q331K^ mice at 16 months of age when compared to NTg and TDP-43^WT^ mice (blue bar compared to orange and green bars in Fig. 4a; *n* = 5, *P* > 0.05). In contrast to protein levels of C5a, C5aR1 mRNA expression was significantly increased by 1.4-fold and 1.2-fold at 10 months of age and by 1.8-fold and 1.6-fold at 16 months of age, when compared to NTg and TDP-43^WT^ mice (blue bar compared to orange and green bars in Fig. 4b; *n* = 5, *P < 0.05, ***P < 0.001, ****P < 0.0001).

We next aimed to determine the cellular localisation of C5aR1 that could explain the increased expression seen in TDP-43^Q331K^ mice. To achieve this, we performed immunolabelling for C5aR1 on the lumbar spinal cord from NTg, TDP-43^WT^ and TDP-43^Q331K^ mice. These sections were immunostained for C5aR1 with specific cellular markers to identify motor neurons (anti-ChAT), astrocytes (anti-GFAP) and microglia (anti-Iba-1). C5aR1 localised to ChAT-positive motor neurons and Iba-1-positive microglia in NTg, TDP-43^WT^ and TDP-43^Q331K^ mice at 16 months of age (white arrows in Fig. 4c–e for motor neurons and Fig. 4f–h for microglia), whereas it was not observed in GFAP-positive astrocytes (Fig. 4i–k). Together, the results above indicate that C5a-C5aR1 signalling could play a role in facilitating microglia activation and phagocytosis ultimately leading to motor neuron death in these animals.

### Dysregulation of complement in the tibialis anterior muscle of TDP-43^Q331K^ mice

In addition to showing altered levels of complement components in the spinal cord of TDP-43^Q331K^ mice, we also investigated the level of major complement components in the TA muscle of TDP-43^Q331K^ mice, as it has been shown previously in hSOD1^G93A^ mice that complement is upregulated in this muscle. To investigate this, we measured the mRNA levels of key components of the complement pathways, which include the classical/lectin (C1qB and C4), alternative (fB) and terminal pathways (C5a and C5aR1), as well as the major complement regulators (CD55 and CD59a) using quantitative real-time PCR and enzyme-linked immunosorbent assay for C5a in TA muscle of TDP-43^Q331K^ mice during disease progression of ALS (3, 10 and 16 months).

**Fig. 5:**
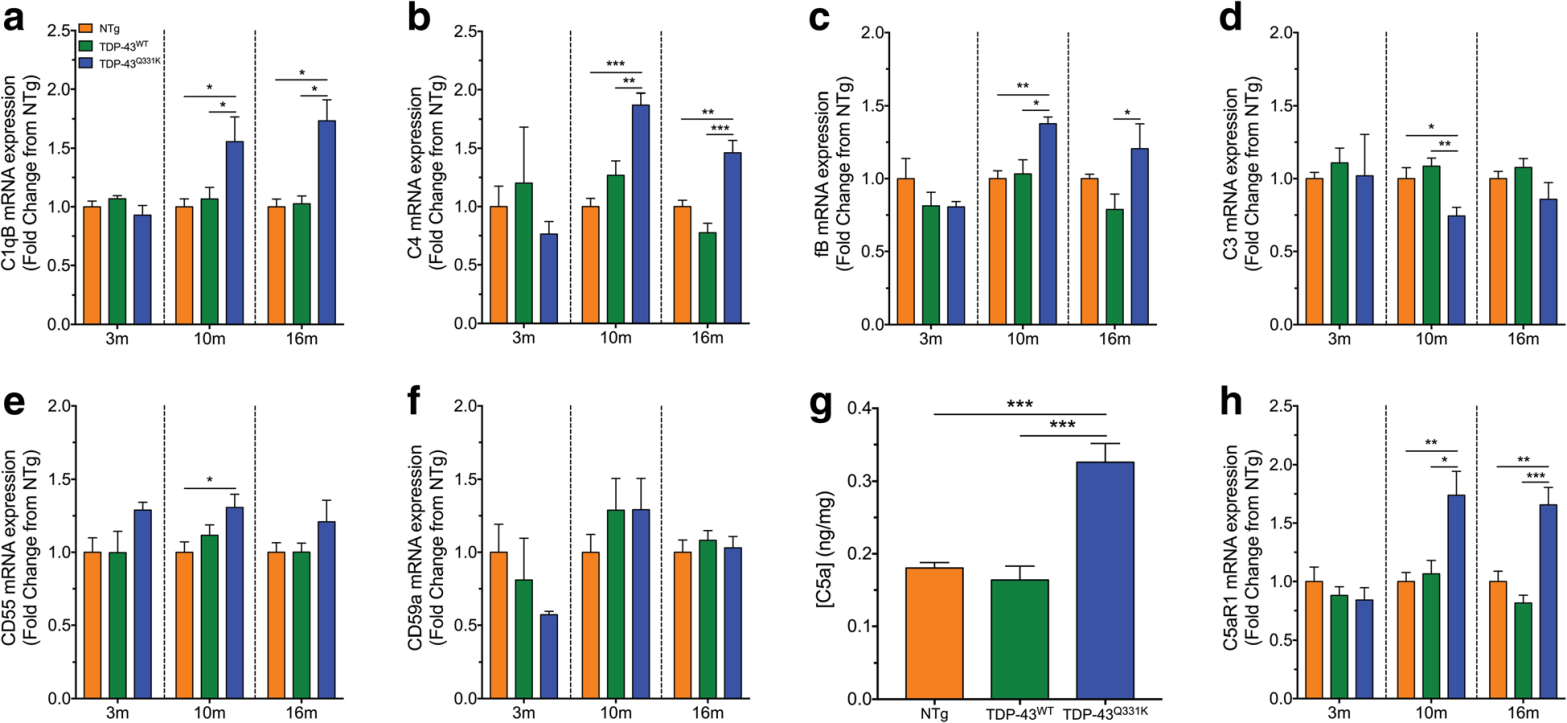
Dysregulation of complement components in tibialis anterior muscle of TDP-43^Q331K^ mice at three different ages of disease progression. **a**–**f** The mRNA expression profiles of the following complement components: C1qB (**a**, classical pathway), C4 (**b**, classical/lectin pathway), fB (**c**, alternative pathway), C3 (**d**, central component), CD55 (**e**, regulator) and CD59a (**f**, regulator) in tibialis anterior muscle of TDP-43^Q331K^ mice (blue bars) relative to non-transgenic (NTg, orange bars) and TDP-43^WT^ (green bars) mice during 3, 10 and 16 months of age. **g** The protein expression of C5a in the tibialis anterior muscle of NTg (orange bars), TDP-43^WT^ (green bars) and TDP-43^Q331K^ (blue bars) mice at 16 months. **h** mRNA expression of C5aR1 in the tibialis anterior muscle of TDP-43^Q331K^ mice relative to age-matched NTg and TDP-43^WT^ mice at three different ages. Data are expressed as means ± SEM (*n* = 5 mice/group, *P < 0.05, **P < 0.01, ***P < 0.001, one-way ANOVA with Tukey’s post hoc test for each age)

C1qB and C4 transcripts were significantly increased by 1.6-fold and 1.9-fold when compared to NTg mice and by 1.5-fold when compared to TDP-43^WT^ mice at 10 months of age, respectively (blue bar compared to orange and green bars in Fig. 5a, b; *n* = 5, *P < 0.05, **P < 0.01, ***P < 0.001). C1qB and C4 transcripts were also increased by 1.7-fold and 1.5-fold when compared to NTg mice and by 1.7-fold and 1.9-fold when compared to TDP-43^WT^ mice at 16 months of age, respectively (blue bar compared to orange and green bars in Fig. 5a, b; *n* = 5, *P < 0.05, **P < 0.01, ***P < 0.001). In addition to C1qB and C4, fB also displayed a marked increase in mRNA levels by 1.4-fold and 1.3-fold at 10 months of age when compared to NTg and TDP-43^WT^ mice, whereas 1.5-fold increase at 16 months of age when compared to TDP-43^WT^ mice (blue bar compared to orange and green bars in Fig. 5c; *n* = 5, *P < 0.05, **P < 0.01). By contrast, C3 was surprisingly decreased by 0.3-fold in TA muscle of TDP-43^Q331K^ mice when compared to NTg and TDP-43^WT^ mice at 10 months of age (blue bar compared to orange and green bars in Fig. 5d; *n* = 5, *P < 0.05, **P < 0.01). The regulator, CD55, was increased by 1.3-fold at 10 months of age in TDP-43^Q331K^ mice when compared to NTg mice (blue bar compared to orange bar in Fig. 5e; *n* = 5, *P < 0.05), while there was no significant change in CD59a in TDP-43^Q331K^ mice when compared to NTg and TDP-43^WT^ controls (Fig. 5f; *n* = 5, *P* > 0.05). Lastly, the terminal complement pathway component, C5a, and its receptor C5aR1 were investigated. The results showed significant increases in C5a protein at 16 months of age by 1.8-fold and 2.0-fold when compared to NTg and TDP-43^WT^ mice respectively (blue bar compared to orange and green bars in Fig. 5g; *n* = 5, ***P < 0.001). C5aR1 mRNA expression was also significantly increased by 1.7-fold and 1.6-fold at 10 months of age and by 1.7-fold and 2.0-fold at 16 months of age when compared to NTg and TDP-43^WT^ controls, respectively (blue bar compared to orange and green bars in Fig. 5h; *n* = 5, *P < 0.05, **P < 0.01, ***P < 0.001). Taken together, these results suggest that dysregulation of the complement system also occurs in the TA muscle of TDP-43^Q331K^ mice, which could contribute to the disease pathology in these animals.

### Motor dysfunction in TDP-43^Q331K^ mice strongly correlates with lumbar spinal cord levels of C1qb, C3 and C5aR1

**Fig. 6:**
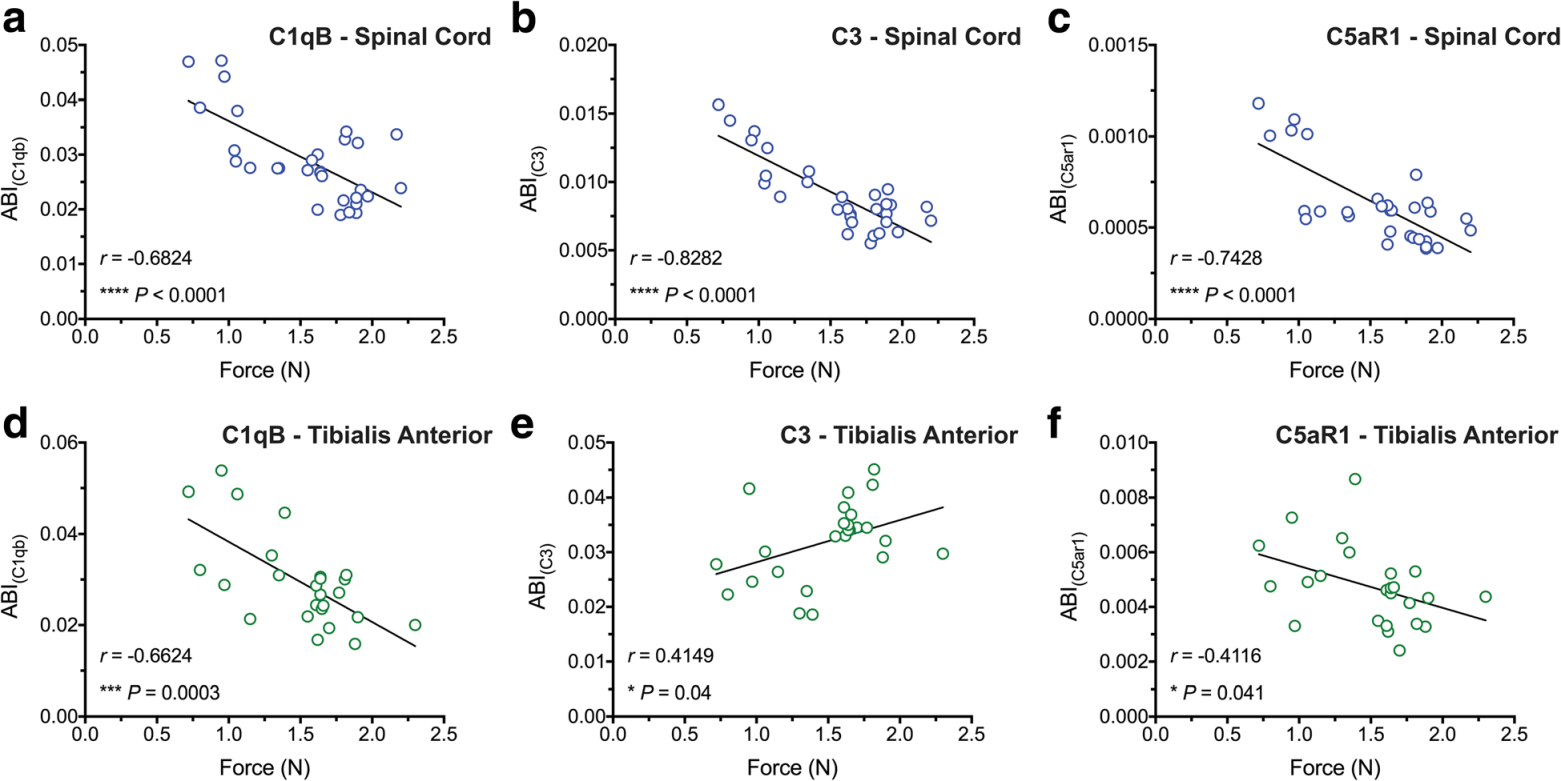
Motor dysfunction in TDP-43^Q331K^ mice strongly correlates with lumbar spinal cord/tibialis anterior muscle levels of C1qB, C3 and C5aR1. A strong negative correlation between lumbar spinal cord mRNA expression of C1qB (**a**, *r* = − 0.6824), C3 (**b**, *r* = − 0.8282) and C5aR1 (**c**, *r* = − 0.7428) with hind-limb grip strength of non-transgenic (NTg), TDP-43^WT^ and TDP-43^Q331K^ mice, where C3 demonstrated the strongest correlation (*n* = 30, ****P < 0.0001, Pearson correlation). A moderate to strong negative correlation between tibialis anterior muscle mRNA levels of C1qB (**d**, *r* = − 0.6624) and C5aR1 (**f**, *r* = − 0.4116) with hind-limb grip strength of NTg, TDP-43^WT^ and TDP-43^Q331K^ mice, whereas a moderate positive correlation between C3 mRNA levels (**e**, *r* = 0.4149) was observed (*n* = 25, *P < 0.05, ***P < 0.001, Pearson correlation). Pearson correlation coefficient was used to measure the strength of the linear relationship between complement mRNA expression and hind-limb grip strength

We next examined if there was any correlation between major complement transcript levels to the hind-limb grip strength of TDP-43^Q331K^ mice. To investigate this aspect, we performed a Pearson correlation to measure the strength of the linear relationship between C1qB, C3 and C5aR1 mRNA levels in the lumbar spinal cord and TA muscle of NTg, TDP-43^WT^ and TDP-43^Q331K^ mice with their hind-limb grip strength. We found a strong negative correlation between lumbar spinal cord mRNA expression of C1qB (*r* = − 0.6824), C3 (*r* = − 0.8282) and C5aR1 (*r* = − 0.7428) to hind-limb grip strength of these animals, with C3 presenting the strongest correlation (*n* = 30, ****P < 0.0001; Fig. 6a–c). Furthermore, a moderate to strong negative correlation between TA mRNA levels of C1qB (*r* = − 0.6624) and C5aR1 (*r* = − 0.4116) to hind-limb grip strength was observed, whereas a moderate positive correlation was identified between C3 mRNA levels (*r* = 0.4149) to hind-limb grip strength (*n* = 25, *P < 0.05, ***P < 0.001; Fig. 6d–f). These results indicate that changes in complement transcript levels in the spinal cord and TA muscle directly correlates with the decrease in hind-limb grip strength (i.e. increased in ALS symptoms) in this ALS model.

## Discussion

The major findings of the current study are that components of the complement system are dysregulated in a transgenic mutant TDP43-based mouse model of ALS. It has now been well documented that the complement cascade is synthesised by neurons, astrocytes and microglia within the central nervous system and is involved in the disease progression of ALS, with evidence from both human patients and rodent models [8]. The present study further adds to this knowledge, demonstrating alteration of mRNA expression in major complement factors including C1qB, C4, fB, C3, C5a, C5aR1 and regulator CD55 in the spinal cord and TA muscles of TDP-43^Q331K^ mice, which is suggestive of a progressive dysregulation of complement in this model. These results are similar to our previous demonstration of complement dysregulation in the spinal cord and TA muscle of hSOD1^G93A^ mice [11, 15], indicating that complement activation occurs in response to motor neuron death and muscle denervation regardless of which ALS-related gene mutation is present. Furthermore, complement activation, and subsequent C5aR1 activation, could be a common mechanism of pathology in most forms of ALS.

The present study provided evidence for the dysregulation of classical/lectin, alternative and central component to all pathways of the complement system in the spinal cord and TA muscle of TDP-43^Q331K^ mice during ALS disease progression. This is in line with numerous studies including our own, where increased C1qB, C4 and C3 was found in the spinal cord of hSOD1^G93A^ mice [11, 15, 22, 23]. This upregulation of C1qB and C3 in the spinal cord could assist in the removal of dying motor neurons via opsonisation through microglia activation during disease progression in TDP-43^Q331K^ mice, similar to what is evident in hSOD1^G93A^ mice [15]. Similarly, we identified upregulation of C1qB, C4 and fB in the TA muscles of TDP-43^Q331K^ mice, however, found downregulation of C3 in the TA muscles of TDP-43^Q331K^ mice, which is contradictory of what is shown in hSOD1^G93A^ mice. The upregulation of C1qB in the TA muscles could assist in the removal of degenerating neuromuscular synapses via phagocytosis during disease progression in TDP-43^Q331K^ mice, similar to what has been shown in hSOD1^G93A^ mice [22]. The reason for the differential expression of C3 in the TA muscle of TDP-43^Q331K^ mice compared with hSOD1^G93A^ mice is unclear, but could be due to the difference in the severity of the disease with TDP-43^Q331K^ mice showing milder ALS-like symptoms compared to hSOD1^G93A^ mice [13, 14].

We additionally showed decreased mRNA expression levels of CD55 in the spinal cord and increased expression levels in TA muscle, which suggests that homeostatic balance of the complement system may be perturbed in TDP-43^Q331K^ mice, leading to the over activation of complement system. These findings support other studies, which have also shown decreased CD55 mRNA levels in the spinal cord, with deficiency in CD55 exacerbating neurodegeneration [15, 22, 24]. Our findings also support previous studies identifying increased CD55 expression in intercostal muscles of ALS patients and TA muscles of hSOD1^G93A^ mice [11, 25]. Upregulation of CD55 at the motor end-plates of ALS patients and hSOD1^G93A^ mice could be a mechanism to protect against high levels of complement activation at the neuromuscular junction. Interestingly, the current study did not demonstrate any changes in the other regulator CD59a in the spinal cord or TA muscle of TDP-43^Q331K^ mice. This is in contrast to previous studies, where CD59a was decreased in the spinal cord of hSOD1^G93A^ mice, while it increased in the intercostal muscle of ALS patients and TA muscle of hSOD1^G93A^ mice [11, 15, 25]. The difference in the expression changes between different models and patients could be attributed to the severity of the disease, as TDP-43^Q331K^ mice show milder ALS-like symptoms compared to hSOD1^G93A^ mice [9, 13–15]. However, further investigation is required into the expression and localisation of membrane attack complex in TDP-43^Q331K^ mice, and its putative correlation with motor neuron loss. Regardless, collectively our data adds further support to the notion that dysregulated complement activity may play an important role in accelerating motor neuron loss and neuromuscular junction denervation, ultimately driving the progression of ALS.

Among the complement activation effector molecules, C5a is considered the most potent peptide, with its signalling through its main receptor C5aR1 having detrimental effects in multiple neurodegenerative diseases, including ALS [9, 10, 12, 26, 27]. To obtain a better understanding of its role in disease progression of ALS, we analysed C5a and its receptor C5aR1 in the spinal cord and TA muscle of TDP-43^Q331K^ mice. C5a protein levels in the spinal cord did not change between NTg, TDP-43^WT^ and TDP-43^Q331K^ mice, whereas it increased in the TA muscles of TDP-43^Q331K^ mice. This is in line with C5a protein levels at mid-symptomatic stage in the spinal cord and TA muscle of hSOD1^G93A^ mice [11], suggesting that enhanced C5a-C5aR1 signalling may affect disease progression of ALS, similar to what has been found in hSOD1^G93A^ mice. This finding is interesting given that C3 mRNA transcript levels were higher in the spinal cord compared to TA muscle in these animals. The reason for the increase in muscle C5a, despite reduced C3 transcript expression is unclear; however, it should be noted that mRNA expression does not directly equate with the degree of complement activation. Furthermore, there is a well-described C3-bypass (extrinsic) complement activation pathway that enables C3-independent cleavage of C5 [28]. Thus, C5a protein levels do not necessarily correlate with C3 mRNA expression within a tissue.

Numerous studies have demonstrated upregulation of C5aR1 within the spinal cord and TA muscles of hSOD1^G93A^ rats and mice, as well as human ALS patients, suggesting that heightened C5a-C5aR1 signalling plays a role in ALS pathology [9–12, 15, 29]. In the present study, we demonstrated that the mRNA expression of C5aR1 in TDP-43^Q331K^ spinal cord and TA muscles is elevated, confirming dysregulation of downstream terminal complement pathway in these animals. This suggests that enhanced C5a-C5aR1 signalling may affect the disease progression of ALS in both spinal cord and TA muscles of TDP-43^Q331K^ mice, similar to what is evident in hSOD1^G93A^ mice [9, 11, 15]. In addition to an increase in C5aR1 in TDP-43^Q331K^ mice, the present study also revealed C5aR1 on motor neurons and microglia in NTg, TDP-43^WT^ and TDP-43^Q331K^ mice. This is in concordance with previous studies showing C5aR1 on motor neurons and microglia in other mouse models of ALS [15, 20], indicating that C5a-C5aR1 signalling could play a role in facilitating microglia activation and motor neuron death regardless of which ALS-related gene mutation is present.

In line with increased complement components in TDP-43^Q331K^ mice, the present study also revealed a strong correlation between C1qB, C3 and C5aR1 mRNA levels in the spinal cord and TA muscles to the hind-limb grip strength of these animals, suggesting that the mRNA levels of complement system could be a direct correlate of motor neuron loss and neuromuscular junction denervation throughout disease progression, that we and others have previously demonstrated in this mouse model [13, 14]. These finding are consistent with increased astrocyte and microglia numbers/activation in these animals, indicating that neuroinflammation could be a good indicator of disease severity and pathology.

## Conclusions

In summary, the current study has demonstrated upregulation of major complement factors, together with decreased levels of the negative complement regulator CD55, in TDP-43^Q331K^ mice. This suggests that complement activation and/or its dysregulation could play an important role in motor neuron loss and neuromuscular junction denervation in this TDP43-based mouse model of ALS. Expression of the C5a receptor, C5aR1, was also upregulated in TDP-43^Q331K^ mice, predominantly due to increased microglial/macrophage C5aR1 expression, and was strongly correlated with resulting motor decline. Taken together, these results indicate that heightened complement activation and enhanced C5aR1 signalling may play a crucial role in pathophysiology of the TDP-43^Q331K^ ALS model, further validating C5aR1 as a potential therapeutic target for all forms of ALS.

## Abbreviations

ALS: Amyotrophic lateral sclerosis
BSA: Bovine serum albumin
DAPI: Diamidino-2-phenylindole
DS: Donkey serum
NTg: Non-transgenic
TA: Tibialis anterior
